# Introduction of RKKY-pMTJ-Based Ultrafast Magnetic Sensor Architecture and Magnetic Multilayer Optimization

**DOI:** 10.3390/s25216793

**Published:** 2025-11-06

**Authors:** Jaehun Cho, June-Seo Kim

**Affiliations:** Division of Nanotechnology, Daegu-Gyeongbuk Institute of Science and Technology (DGIST), Daegu 42988, Republic of Korea

**Keywords:** tunnel magnetoresistance, magnetoresistance sensor, interlayer exchange coupling, Ruderman–Kittel–Kasuya–Yosida (RKKY) interaction, micromagnetic simulations, magnetic multilayer optimization, ultrafast switching

## Abstract

A state-of-the-art tunnel magnetoresistance (TMR) sensor architecture, which is based on the perpendicularly magnetized magnetic tunnel junction (pMTJ), is introduced and engineered for ultrafast, high thermal stability, and linearity for magnetic field detection. Limitations in high-frequency environments, stemming from insufficient thermal stability and slow recovery times in conventional TMR sensors, are overcome by this approach. The standard MRAM structure is modified, and the Ruderman–Kittel–Kasuya–Yosida (RKKY) interaction is employed to give a strong, internal restoring torque to the storage layer magnetization. Sensor linearity is also ensured by this RKKY mechanism, and rapid relaxation to the initial spin state is observed when an external field is removed. The structural and magnetic properties of the multilayer stack are experimentally demonstrated. Robust synthetic antiferromagnetic (SAF) coupling is confirmed by using polar MOKE spectroscopy with an optimal Ru insertion layer thickness (0.6 nm), which is essential for high thermal stability. Subsequently, an ultrafast response of this TMR sensor architecture is probed by micromagnetic simulations. The storage layer magnetization rapidly recovers to the SAF state within an ultrashort time of 5.78 to 5.99 ns. This sub-6 ns recovery time scale suggests potential operation into the hundreds of MHz range.

## 1. Introduction

TMR (Tunnel Magneto-Resistance) sensors, characterized by exceptionally high sensitivity and low power consumption, are widely employed for the precise detection of weak magnetic fields. In the automotive sector, they are used in applications requiring high reliability, such as current sensing in electric vehicle (EV) battery systems, steering angle measurement, and wheel-speed detection in anti-lock braking systems (ABSs) [1,2,3,4]. Beyond transportation, TMR sensors play a pivotal role in smart manufacturing, where they enable precise control of motor position and angular displacement in robotic arms and automated equipment [5]. Owing to their superior performance compared to conventional Hall sensors and AMR (Anisotropic Magneto-Resistance) or GMR (Giant Magneto-Resistance) sensors, TMR sensors are rapidly expanding their influence across advanced fields that demand high-precision magnetic sensing [6].

Conventional TMR sensors typically employ CoFeB layers with thicknesses of only a few nanometers, which inherently exhibit strong in-plane magnetic anisotropy [6,7,8]. Consequently, the magnetization of the free layer mostly rotates within the plane relative to the pinned layer, thereby modulating the tunneling currents. Since the free layer magnetization is stabilized primarily in the in-plane direction, the sensor characteristics are generally optimized for detecting in-plane magnetic fields. Such devices are commonly referred to as “in-plane magnetized MTJ sensors” (or simply “in-plane TMR sensors”) [8].

In contrast, the perpendicularly magnetized magnetic tunnel junction (pMTJ) architecture offers several significant advantages for sensor development, but practical implementation as sensor devices has remained limited due to fabrication challenges. In pMTJs, the magnetization is aligned perpendicular to the film plane (out-of-plane), which facilitates device miniaturization and high integration density while also enhancing the efficiency of spin-transfer (STT) and spin–orbit torques (SOTs). The perpendicular configuration further enables the design of response characteristics distinct from those of in-plane field sensors. Moreover, because the fabrication processes of pMTJs are largely compatible with those of magneto-resistive random-access memory (MRAM), this architecture holds strong potential for integration with semiconductor circuits [9,10]. Achieving this crucial perpendicularly magnetized structure using Perpendicular Magnetic Anisotropy (PMA) relies on specific material engineering strategies. The most common method, interfacial PMA, utilizes the strong spin–orbit coupling at the interface between a heavy metal and a ferromagnetic layer, as seen in the Pt/Co, Pd/Co structures [11,12,13,14]. Co/Pt superlattice structures are candidates for enhancing the PMA energy [15,16]. Alternatively, interface bonding with an oxide layer, such as in the highly relevant Ta/CoFeB/MgO structure, is utilized [17,18,19]. Alternatively, bulk PMA can be achieved by utilizing the intrinsic crystal structure of materials like L_10_-ordered alloy, which consists of Co, Fe and Pt, Pd such as FePt or FePd alloys [20,21], which possess a large, intrinsic magnetocrystalline anisotropy suitable for high-density, thermally stable applications.

The main challenge in developing pMTJ-based TMR sensors is in the fact that the MRAM architecture cannot be directly used. Conventional MRAM structures consist of a storage layer (SL) for binary information and a reference layer (RL) with fixed magnetization. The SL thickness is optimized to maximize the magnetoresistance (MR) ratio. However, TMR sensors are always required to operate over a wide temperature range (typically −40 °C to +80 °C), and the ultrathin SL used in MRAM shows insufficient thermal stability [22,23]. Therefore, it is difficult to ensure a sufficient retention time of the TMR sensor in a higher temperature region. To overcome this limitation, radically new MRAM architecture should be implemented for high thermal stability TMR sensor development.

In this study, we propose a modified MRAM architecture for next-generation TMR sensors. The key difference from conventional MRAM is to employ an interlayer exchange coupling (IEC), specifically the Ruderman–Kittel–Kasuya–Yosida (RKKY) interaction of appropriate strength, to stabilize the magnetization of the SL [24]. This architecture offers two major advantages: (i) under the influence of IEC or RKKY coupling, the SL acquires an angle that depends on the strength of the external magnetic field, thereby ensuring linearity in the TMR sensor response; and (ii) when the external magnetic field is reduced, the SL magnetization is driven back to its initial state through the restoring effect of IEC or RKKY coupling [25]. Previous studies on SAF–pinned MTJ devices have primarily utilized interlayer exchange coupling to stabilize the reference layer in magnetic-memory applications, thereby minimizing dipolar interaction with the free layer. In contrast, the RKKY-coupled pMTJ proposed in this work employs the coupling as an active restoring mechanism for the storage layer itself. This functional shift transforms the conventional passive pinning role into a dynamic stabilization scheme that enables spin-torque-free, field-driven operation. This mechanism allows rapid recovery between synthetic-ferromagnetic and synthetic-antiferromagnetic states within a few nanoseconds, revealing the physical basis for the achievable hundreds-of-MHz-level dynamic response discussed in this work. Consequently, the proposed structure bridges memory- and sensor-type MTJs, offering ultrafast recovery while preserving thermal robustness.

As described above, the pMTJ-type TMR sensor exhibits a high sensitivity to variations in the magnetic field while also possessing a natural tendency for the SL magnetization to return to its initial state. To investigate the dynamic response and magnetization behavior of the sensor, micromagnetic simulations are systematically performed. The results reveal that, when the external magnetic field is removed, the SL magnetization relaxes back to its initial state within 1–6 ns depending on the applied magnetic fields. This result suggests that pMTJ-type TMR sensors hold strong potential for applications as high-speed position and angle sensors, as well as in ultrafast leakage current detection.

Finally, magnetic multilayer films were fabricated and optimized to realize the pMTJ-type TMR sensor. To achieve perpendicular magnetic anisotropy, Pt/Co multilayers were employed, while Ru was introduced as an insertion layer to form a synthetic antiferromagnetic (SAF) structure via IEC or RKKY interactions. As a result, the optimal Ru thickness for implementing the pMTJ-type TMR sensor was identified, and a complete magnetic multilayer stack was fabricated to evaluate and verify its performance as a TMR sensor.

## 2. Sensor Architecture and Working Principle

**Figure 1:**
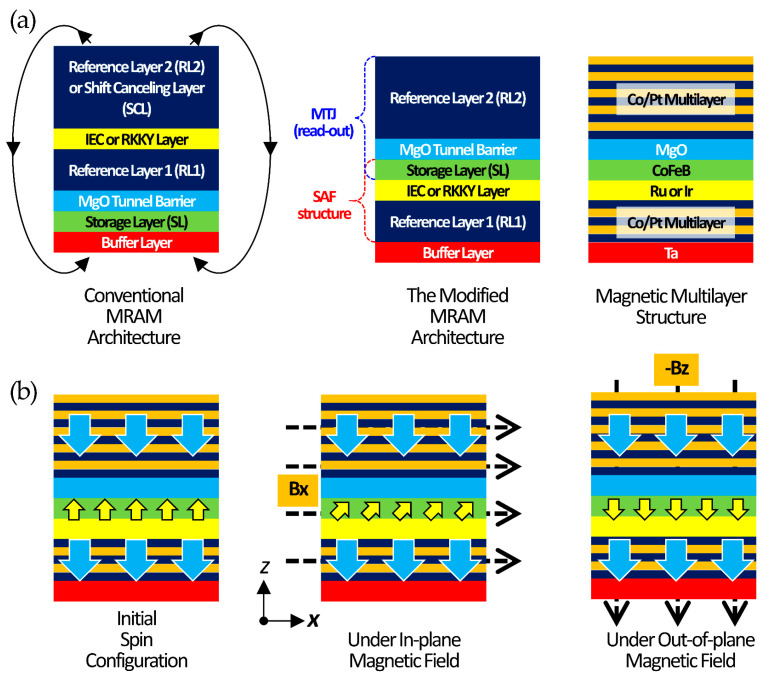
RKKY-pMTJ-based MTJ sensor architecture and working principle: (**a**) conventional MRAM architecture, the modified MRAM architecture, and magnetic multilayer structure. (**b**) Initial spin configuration without external field, RKKY-pMTJ-based TMR sensor working principle with in-plane and out-of-plane applied magnetic fields.

First, the conventional MRAM architecture is depicted in Figure 1a. It consists of an SL for information retention and an ultrathin MgO tunnel barrier. In this architecture, two RLs are required, which are coupled through a thin IEC or RKKY interaction to form a synthetic antiferromagnetic (SAF) configuration. The role of RL1 is essential, as it completes the magnetic tunnel junction. However, since the RLs are typically composed of Pt/Co multilayers with very strong magnetization, substantial magnetic stray fields are generated. These stray fields induce a shift in the hysteresis loop of the SL. To solve this problem, a much thicker RL2 is introduced so that the stray field from RL2 compensates for that generated by RL1. For this reason, RL2 is often referred to as the shift-canceling layer (SCL).

To adapt the conventional MRAM architecture for use as an ultrasensitive TMR sensor, a modified structure is devised (Figure 1a). The key difference is that the SAF configuration is formed between RL1 and the SL, thereby providing the SL with strong restoring torque. In this scheme, RL2 assumes only the function of the reference layer, which is originally played by RL1 in conventional MRAM. The magnetic multilayer structures employed in this study are shown in Figure 1a. The reference layers are composed of Co/Pt multilayers, while the SL is realized either as a CoFeB single layer or as a Pt/Co/CoFeB multilayer. Ru is used as the insertion layer to induce IEC or RKKY interactions, and the exact SL configuration is determined by the strength of these couplings.

Figure 1b illustrates the operating principle of the modified MRAM-architecture-based TMR sensor. In the initial spin configuration, the two reference layers are aligned parallel to each other, while the SL is antiparallel due to the SAF structure. When an in-plane magnetic field, B_x,_ is applied, the SL tilts by a finite angle within the plane, and the relative angle between the SL and RL determines the TMR value. In this mode, the device functions as an analog-type TMR sensor. In contrast, when an out-of-plane magnetic field exceeding a certain threshold is applied, magnetization reversal of the SL occurs, and the device operates as a digital-type TMR sensor. Importantly, in both cases, once the external field is removed, the SAF structure provides a strong restoring force, which enables the system to rapidly return to its initial spin configuration. As shown in Figure 1b, under a small in-plane bias field, the SAF alignment exhibits a quasi-linear response due to slight canting of the magnetic moments of SL. This regime can serve as a linear sensing mode, where the resistance change arises from angular deviation between the layers. In this work, the out-of-plane recovery dynamics are emphasized to demonstrate the ultrafast RKKY-driven stabilization, while the in-plane response confirms the structural versatility of the proposed architecture.

## 3. Experimental Methods

In this study, the multilayer structure of RL1 was designed as Pt (4.0)/[Co (0.6)/Pt (0.2)]_5_ (units in nm) deposited on a 100 nm-thick thermally oxidized SiO_2_ substrate with a Ta (4.0) buffer layer. On top of this stack, a wedge-type Ru insertion layer with thickness varying from 0 to 5.6 nm was deposited by using an automatically motorized programmable shadow mask. The multilayer structure of the SL was chosen as Pt (0.2)/[Co (0.6)/Pt (0.9)]_2_/Co (0.6)/Co_20_Fe_60_B_20_ (0.3), where the ultrathin CoFeB layer was incorporated to promote MgO deposition and ensure well-matched crystallinity. Since RL2 serves only as the readout element in the RKKY-pMTJ TMR sensor, it was not deposited in this fabrication process, and a Ta (4.0) capping layer was placed above the MgO barrier. The base pressure during deposition was maintained at approximately 1 × 10^−9^ Torr, and the Ar working pressure was fixed at 2 mTorr. All samples are post-annealed at 240 °C for 1 h under an out-of-plane magnetic field of 300 mT.

**Figure 2:**
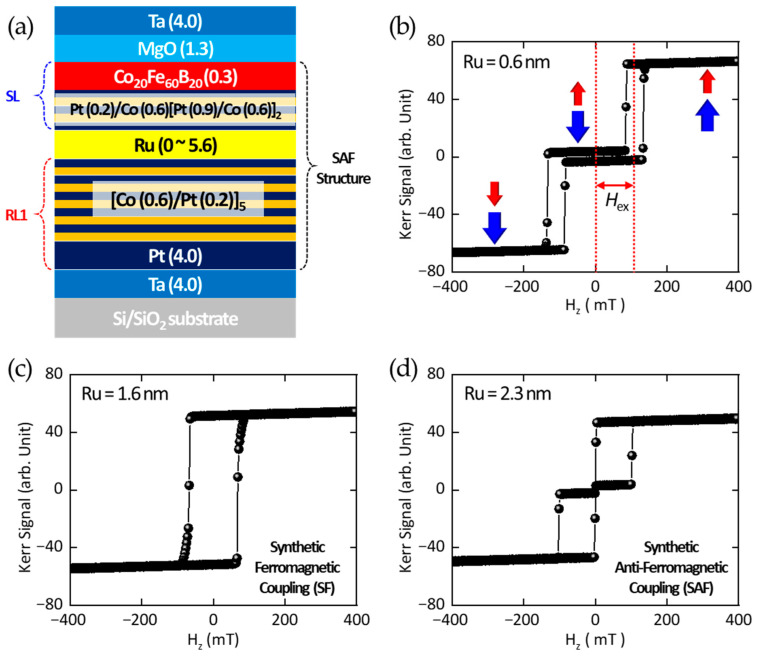
(**a**) The magnetic multilayer structure of RKKY-pMTJ TMR sensor (thickness in nanometers). (**b**) The magnetic hysteresis loop of the SAF coupled structure with Ru = 0.6 nm. Red and blue arrows indicate the directions of the magnetizations of SL and RL, respectively. The red dotted lines are indicating the exchange field, *H*_ex_. (**c**) The magnetic hysteresis loop of the synthetic ferromagnetically (SF) coupled structure with Ru = 1.6 nm. (**d**) Another SAF coupled structure with Ru = 2.3 nm.

The next step is to employ magneto-optical Kerr effect (MOKE) spectroscopy to measure the out-of-plane magnetic hysteresis loops as a function of Ru thickness and to determine the corresponding exchange field (H_ex_) in the SAF structure. The out-of-plane hysteresis loops for varying Ru thicknesses were acquired using a commercial NanoMOKE3 system (Durham Magneto-Optic Ltd., Toft, Cambridge, UK). Figure 2b presents the magnetic hysteresis loop of the RKKY-pMTJ-based TMR sensor for the case of Ru = 0.6 nm. The red and blue arrows indicate the magnetization orientations of RL1 and SL, respectively. Without external magnetic field in the *z*-direction, i.e., *H*_z_ = 0 mT, the Kerr signal nearly vanishes because the magnetizations of SL and RL1 are antiparallel, resulting in signal cancelation. This observation confirms the robust formation of the SAF structure.

## 4. Results and Discussion

### 4.1. Magnetic Multilayer Structure for RKKY-pMTJ-Based TMR Sensor

In this subsection, the magnetic multilayer structure of the RKKY-pMTJ-based TMR sensor is introduced. Particular attention is given to the design of RL1 and the SL, and experimental verification is carried out to confirm whether the SAF configuration is established as a function of the Ru insertion layer thickness.

**Figure 3:**
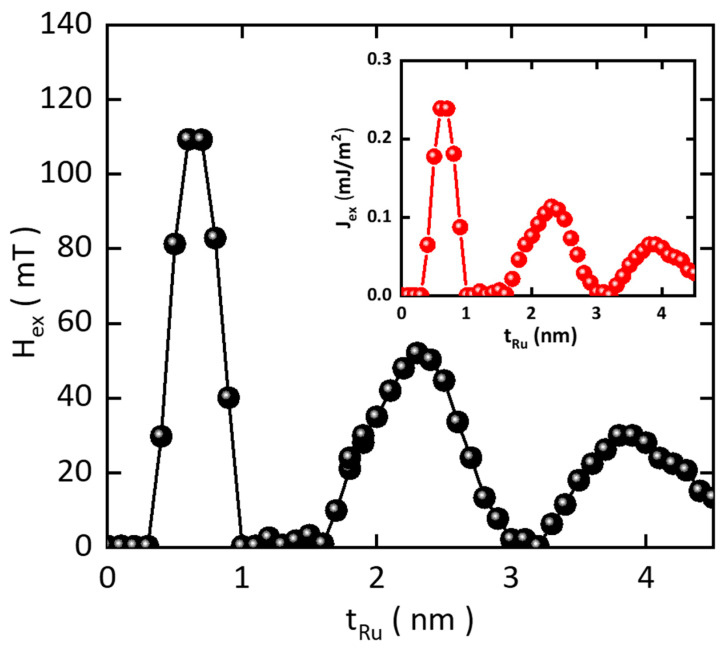
Insertion layer thickness-dependent strength of RKKY interaction, Ru thickness-dependent H_ex_ measurements (Black filled circles) (inset). Conversion curve of *H*_ex_ to RKKY exchange energy (*J*_ex_) (Red filled circles).

Due to the stray fields originating from RL1 and SL, a difference arises between the switching fields observed during the increasing and decreasing field sweeps in the magnetic hysteresis loops. The arithmetic mean of these two switching fields is defined as the exchange field (H_ex_). For the case of H_ex_ = 0, the individual magnetic multilayer structures are ferromagnetically coupled, a state referred to as SF coupling. Figure 2c shows the magnetic hysteresis loop measured at a Ru thickness of 1.6 nm, where clear evidence of SF coupling is observed. In contrast, as the Ru thickness is further increased, the SAF configuration reappears. Figure 2d presents the hysteresis loop at a larger Ru thickness, where SAF coupling is observed with Ru = 2.3 nm. To ensure reproducibility, each measurement was repeated three times on separate regions of the same sample, and the variations in coercive and exchange fields were within ±3%. The instrumental uncertainty of the MOKE setup was approximately ±2 mT, and representative data are presented in Figure 2b and Figure 3.

### 4.2. Ru Thickness-Dependent RKKY Interaction and Periodicity Measurements

By employing a wedge-type thin film growth technique with a programmable shadow mask, the dependence of the exchange field (*H*_ex_) on the insertion layer (Ru) thickness can be quantitatively examined. Polar MOKE measurements are systematically performed at equally spaced positions using a motorized programmable sample holder, and each hysteresis loop is averaged over more than ten cycles to ensure data reliability. The focused laser spot is translated with a step size of 1 μm, corresponding to an incremental Ru thickness of approximately 0.1 nm.

Figure 3 presents the experimental results of *H*_ex_ as a function of *t*_Ru_. The data exhibit a pronounced oscillatory decay with a period of approximately 1.0 nm, showing three well-defined SAF maxima at *t*_Ru_ = 0.6, 2.3, and 3.9 nm, consistent with earlier reports [26,27]. The maximum exchange field reaches about 110 mT at *t*_Ru_ = 0.6 nm. The nearly constant spacing between consecutive maxima—1.7 nm between the first and second peaks and 1.6 nm between the second and third—indicates that the coupling oscillations are governed by a well-defined electronic structure of Ru. According to the extended theoretical model [28,29], the oscillation period Λ1 of SAF coupling is given by 2π/*q*_s_, where *q*_s_ is the spanning wave vector connecting two antiparallel points on the Fermi surface. The experimentally observed Λ1 agrees closely with the period predicted from extremal Fermi surface spanning vectors of Ru (0001) [30]. Because Pt and Ru have different crystal structures and distinct Fermi surface topologies, the Λ1-periodic coupling cannot propagate effectively through the Pt spacer layer [31].

The interlayer exchange coupling strength *J*_ex_ is evaluated using the relation [32,33]:

*J*_ex_ = *M*_s_*tH*_ex_, (1)

where *M*_s_ is the saturation magnetization, and *t* is the thickness of the upper ferromagnetic layer [13,14]. The inset of Figure 3 shows *J*_ex_ as a function of t_Ru_. With a saturation magnetization of *M*_s_ = 1040 kA/m (measured by vibrating sample magnetometer, VSM) and a nominal ferromagnetic thickness of *t* = 2.1 nm, a strong coupling strength of *J*_ex_ ≈ 0.24 mJ/m^2^, which is directly proportional to *H*_ex_ ≈ 110 mT, is obtained at *t*_Ru_ = 0.6 nm. This confirms that strong and tunable IEC can be achieved by optimizing the Ru thickness, a critical requirement for the stable operation of RKKY-pMTJ-based TMR sensors.

### 4.3. Structural Analysis of RKKY-pMTJ-Based Ultrafast TMR Sensor via Transmission Electron Microscopy

**Figure 4:**
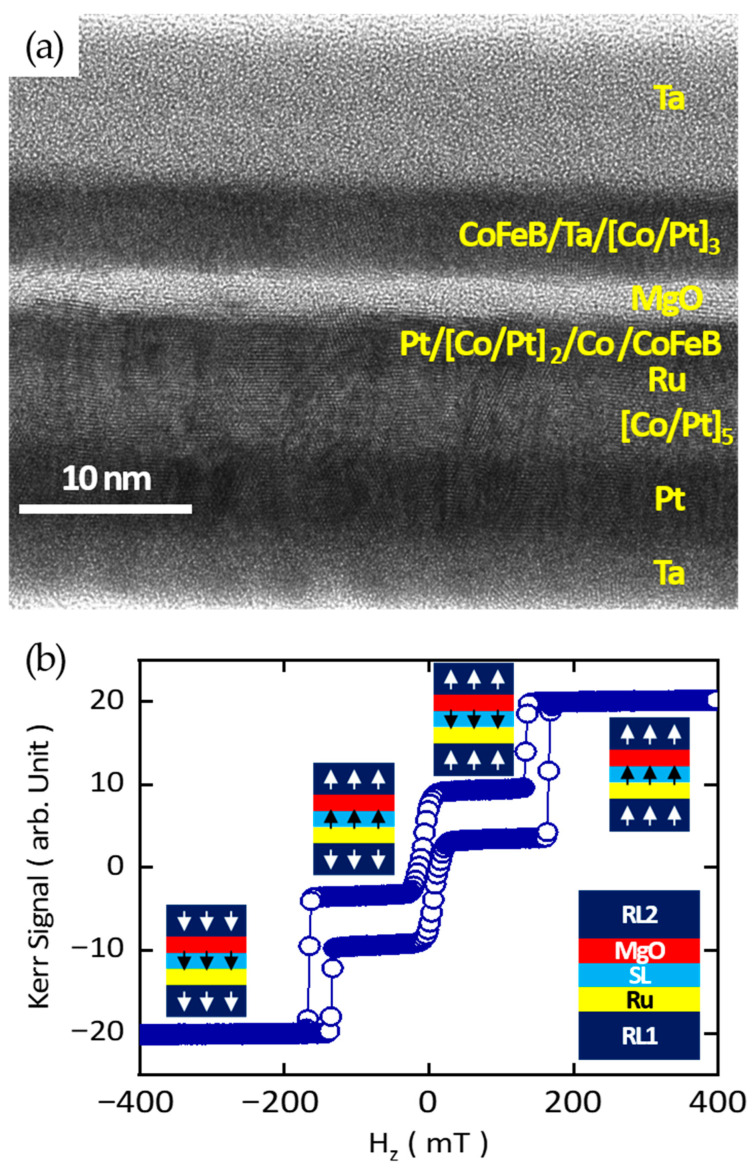
(**a**) Cross-sectional transmission electron microscopy (image) of RKKY-pMTJ-based ultrafast TMR sensor architecture with full magnetic multilayer stack, (**b**) polar MOKE measurement as a function of out-of-plane magnetic field (H_z_). The full magnetic multilayer stack information and the magnetization configurations of all layers and each step in magnetic hysteresis loop are visualized. White and Black arrows indicate the directions of the magnetizations of RL1, RL2, and SL, respectively.

Finally, the complete magnetic multilayer stack of the RKKY-pMTJ-based TMR sensor architecture (referred to as “the modified MRAM architecture”), including the second reference layer (RL2), is fabricated, and cross-sectional transmission electron microscopy (TEM) is carried out to evaluate the structural quality (Figure 4a). The RL2 structure is designed as Co_20_Fe_60_B_20_/Ta/[Co/Pt]_3_, as illustrated in Figure 4a. Remarkably, despite the fact that SL, RL1, and RL2 are all based on Pt/Co superlattices, the multilayers exhibit well-defined crystalline ordering. The MgO tunnel barrier also grows uniformly without detectable defects, confirming the robustness of the deposition process.

The full stack comprises three individual magnetic layers that are intricately coupled. Notably, because SL and RL2 are separated by the MgO barrier, a strong IEC is also present across the tunnel barrier. Nevertheless, the magnetic hysteresis loops exhibit well-defined steps and pronounced squareness. This behavior indicates that each of the three magnetic layers retains its distinct functionality within the multilayer architecture while still contributing to the overall stability of the device. The observation of such sharp magnetic transitions demonstrates both the high structural quality of the multilayer films and the effective magnetic decoupling among the layers. Furthermore, the results are consistent with the existence of proximity-induced magnetic moments at the Co/Pt superlattices, which are known to enhance interfacial anisotropy and play a crucial role in stabilizing the perpendicular magnetization of the p-MTJ sensor.

Although this architecture employs a TMR junction for electrical readout, its core functionality is governed by the RKKY-mediated interlayer exchange coupling rather than by the tunnel-barrier transport properties. The storage-layer magnetization is restored by the intrinsic exchange torque, not by spin-transfer or voltage-driven effects, making the TMR ratio and field sensitivity secondary parameters in this proof-of-concept study. The current work therefore focuses on establishing the magnetic stability and dynamic response enabled by RKKY coupling, which define the fundamental operation speed and robustness of the device. For practical implementation, a TMR-based readout structure can be integrated without altering the magnetic design; based on comparable Ta/CoFeB/MgO systems, the expected TMR ratio is ≈80–120%, and the field sensitivity ≈ 0.1–0.2%/Oe [7,17]. The immediate future work will focus on the microfabrication and electrical characterization of TMR elements using the optimized stack. Direct electrical characterization, including TMR ratio, sensitivity, and linearity range, will be conducted in future work to experimentally validate the device-level performance.

### 4.4. Micromagnetic Simulations for Ultrafast TMR Sensor Response: Magnetic Field-Driven SL Reversal and Recovery Time Calculations

In this subsection, micromagnetic simulations are systematically performed to investigate the dynamic response of the RKKY-pMTJ–based TMR sensor. In particular, quantitative calculations are carried out to determine the characteristic switching time required for the sensor to transform from the SAF configuration in its initial equilibrium state to the SF configuration under the influence of an external magnetic field, as well as the relaxation time needed for the system to return to its initial state once the field is removed. These analyses provide critical insight into the temporal response and stability of the device, which are key performance metrics for high-speed sensor applications.

**Figure 5:**
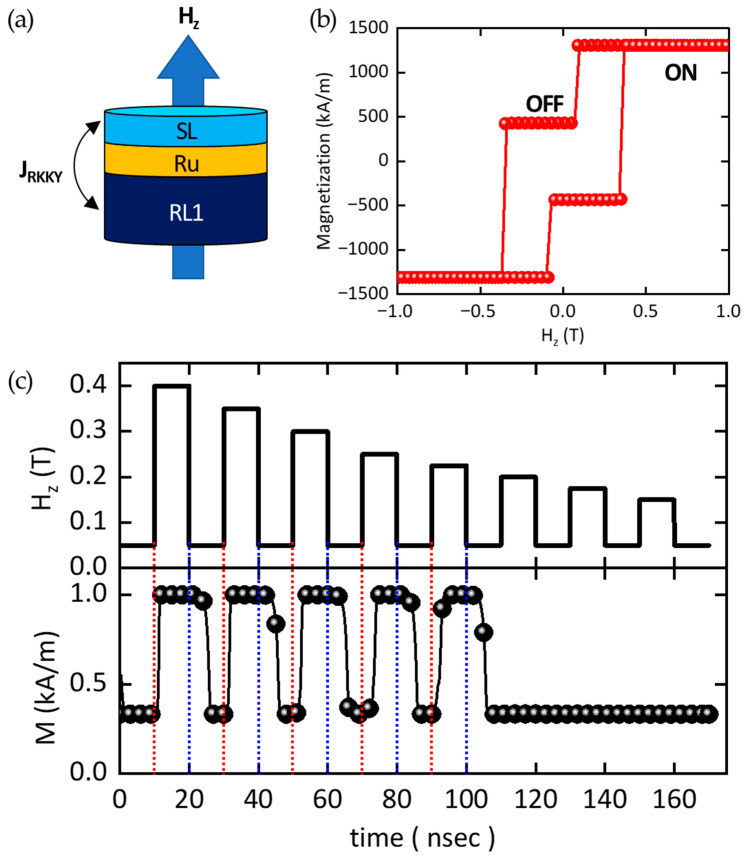
Micromagnetic simulations for ultrafast TMR sensors: (**a**) Schematic configuration of modeling geometry. *J*_RKKY_ between SL and RL1 is expressed. (**b**) Magnetic hysteresis as a function (Red filled circles) of H_z_. (**c**) Top image: the profile of the out-of-plane magnetic field pulse as a function of simulation time (Black solid line). The amplitude of *H*_z_ is varied from 0.4 T to 0.15 T with a 0.05 T step. Bottom image: The magnetization profile of the modeling system as a function of simulation time(Black filled circles). The blue and red dashed lines mark the onset and termination of each magnetic field pulse, respectively.

Figure 5a presents the schematic configuration employed in the modeling. Since the tunnel barrier and RL2 do not directly contribute to the working principle of the sensor, they are excluded from the simulation for computational efficiency and conceptual clarity. Consequently, the modeled structure consists of RL, the insertion layer, and SL, where SL and RL are coupled via RKKY interaction. The finite-difference micromagnetic solver MuMax3 is performed [34], and all material parameters used in the simulations are summarized in Table 1.

In the simulation, both the thickness of RL1 and SL and the interlayer spacing are fixed at 1 nm, while the lateral dimension of each layer is set to 1 μm × 1 μm. A discretization cell size of 1 × 1 × 1 nm^3^ is employed to ensure accurate resolution of the micromagnetic features. The initial magnetic configuration corresponds to a SAF state, since the interlayer exchange coupling constant is *J*_RKKY_ = −1.2 mJ/m^2^ (negative sign indicating antiferromagnetic coupling). An external magnetic field is applied in the out-of-plane direction, denoted as H_z_.

Figure 5b presents the calculated magnetic hysteresis loop of the modeled system. The magnetization is evaluated while sweeping the external field in increments of 0.01 T (10 mT) in both increasing and decreasing directions. In the initial state, the net magnetization does not converge to zero but stabilizes at approximately 430 kA/m. This residual value originates from the imbalance between the saturation magnetizations of RL1 and SL, combined with the fact that the magnitude of *J*_RKKY_ is not sufficiently large to enforce perfect compensation. As H_z_ increases, the magnetization of the SL undergoes reversal, defining the coercive field (*H*_c_), which is determined to be approximately 360 mT.

Finally, the ultrafast switching dynamics of the RKKY-pMTJ-based TMR sensor architecture, hereafter referred to as the modified MRAM structure, are modeled (see Figure 5c). The schematic in the upper panel illustrates the application of an out-of-plane magnetic field pulse. Both the waiting time and the duration time of each pulse are fixed at 10 ns, while the pulse amplitude is systematically reduced from 400 mT in decrements of 50 mT. The simulations are performed down to a pulse amplitude of 150 mT, corresponding to a total simulation time of 170 ns. The blue and red dashed lines mark the onset and termination of each magnetic field pulse, respectively. The “ON” and “OFF” labels indicate the logical states of the TMR sensor corresponding to high- and low-resistance configurations. For the analysis of the simulation results, the operational states of the TMR sensor are defined as follows: (i) During the SAF-to-SF transition, the maximum magnetization reaches approximately 1.0, and the sensor is considered to be in the ‘ON’ state when *M*_s_ exceeds 0.9. (ii) During the SF-SAF relaxation process, the minimum magnetization is approximately 0.3, and the sensor is regarded as being in the ‘OFF’ state when *M*_s_ falls below 0.4.

Table 2 summarizes the fabrication tolerance and scalability of representative MR sensor technologies, highlighting their underlying magnetic mechanisms and contrasting them with the proposed RKKY-pMTJ architecture. AMR sensors use the anisotropic resistance of NiFe films under magnetization rotation; although easy to fabricate, their performance degrades with pattern size and alignment variation. GMR sensors employ spin-dependent scattering in Co/Cu multilayers, achieving higher output but requiring atomic-scale interface smoothness. TMR sensors utilize quantum tunneling through an MgO barrier between ferromagnetic electrodes, yielding large magnetoresistance yet demanding sub-Å barrier-thickness control. In contrast, the RKKY-pMTJ adopts a perpendicular synthetic–antiferromagnetic structure where the restoring torque originates from interlayer exchange coupling rather than geometric precision or barrier uniformity.

This coupling-driven mechanism allows a broader functional layer thickness margin (±17%, ≈±0.1 nm Ru-spacer variation) and supports sub-100 nm device scaling while maintaining thermal stability and ultrafast magnetic recovery. As summarized in Table 2, the RKKY-pMTJ design provides a clear fabrication advantage because its functional stability depends on interlayer coupling rather than on geometric or barrier uniformity. The coupling-based restoring mechanism allows a wider process margin and supports sub-100 nm device scaling, enabling dense integration and consistent performance even under process variation.

From a physical perspective, this modeling framework enables the identification of the critical field amplitude required to trigger reliable magnetization reversal within the SAF–SF transition. The gradual reduction in the pulse amplitude allows one to quantify the threshold field below which the sensor fails to switch within the prescribed timescale. The results highlight the inherent trade-off between switching speed and energy efficiency, since stronger pulses ensure faster transitions, while weaker pulses approach the operational limit of deterministic switching. This analysis therefore provides direct insight into the feasibility of ultrafast operation in practical RKKY-pMTJ-based sensor architectures.

The lower panel of Figure 5c presents the real-time dynamic response of the SL magnetization under applied magnetic field pulses, where the magnetization is recorded with a fixed time step of 0.01 ns (10 ps). Two distinct temporal regimes are particularly relevant for analyzing the magnetization dynamics of the TMR sensor.

In the first regime, corresponding to the onset of a magnetic field pulse, the SL magnetization overcomes the antiferromagnetic interlayer exchange (*J*_RKKY_) and attempts to align with the external field direction. The switching time required for this process is defined as *t*_SAF→SF_. As expected, stronger field amplitudes result in shorter switching times, since the external torque more efficiently drives the magnetization across the energy barrier imposed by J_RKKY_ coupling. As summarized in Table 3, *t*_SAF→SF_ increases gradually from 1.39 ns to 2.94 ns as the field amplitude decreases.

In the second regime, corresponding to the termination of the magnetic field pulse, the system undergoes a relaxation process that restores the initial SAF configuration. The characteristic time of this process, *t*_SF→SAF_, exhibits an intriguing trend: it decreases slightly as the field amplitude is reduced. This behavior arises because weaker field amplitudes produce a smaller net magnetization in the transient SF state, thereby lowering the effective relaxation energy barrier. Nevertheless, the variation of *t*_SF→SAF_ is relatively small, ranging only from 5.99 ns to 5.78 ns, indicating that the recovery dynamics are largely governed by the intrinsic interlayer exchange field rather than the external field history. Also, it is noteworthy that the amplitudes of the magnetic field pulses are insufficient to trigger the SAF-SF transition when the applied magnetic field amplitude is below 200 mT (See Figure 5c). The relaxation time *t*_SF→SAF_ extracted from micromagnetic simulations is approximately 5.8–6.0 ns, representing the interval required for the storage-layer magnetization to return from the transient synthetic–ferromagnetic state to the synthetic–antiferromagnetic equilibrium after the external field pulse is removed. This timescale sets the intrinsic response limit of the RKKY-coupled pMTJ element. By taking f≈1tSF→SAF, the corresponding characteristic frequency is on the order of 1.7 × 10^8^ s^−1^ (≈170 MHz). This indicates that the proposed architecture can in principle sustain sub-10 ns, self-restoring operation driven purely by interlayer exchange coupling. We note that this value reflects the device-level magnetic dynamics of the multilayer stack itself.

From a physical perspective, these findings highlight the asymmetric nature of field-driven switching and relaxation processes in the RKKY-pMTJ architecture. While the forward transition (SAF → SF) is strongly field-dependent, the backward recovery (SF → SAF) is primarily stabilized by intrinsic coupling, ensuring robust return to the equilibrium configuration. This asymmetry underlines both the ultrafast responsiveness and inherent stability of the proposed sensor architecture.

## 5. Conclusions

In summary, this research validates the RKKY-pMTJ as a transformative pathway for magnetic sensing. The comprehensive study first established the structural integrity and magnetic coupling of the multilayer stack via TEM and MOKE analyses, pinpointing the 0.6 nm Ru interlayer as optimal for maximum SAF strength. This foundational work ensured the material system possessed the necessary thermal robustness and strong IEC needed for deterministic operation. Subsequently, dynamic micromagnetic simulations provided definitive proof of concept, showing that the RKKY-stabilized SL magnetization rapidly returns to its stable state in less than 6 ns. This ultrafast recovery and the inherent stability derived from the internal RKKY restoring force represent a significant scientific achievement in high-speed spintronic sensor design. The successful demonstration of a design principle that decouples high-speed operation from dependence on complex external control signals marks a major advancement in the field. In addition to these qualitative findings, this study quantitatively confirmed an RKKY exchange energy of approximately 0.24 mJ/m^2^ at a Ru-spacer thickness of 0.6 nm through systematic IEC analysis. Micromagnetic simulations further verified that the storage-layer magnetization returns to the synthetic–antiferromagnetic equilibrium within ≈6 ns, corresponding to an intrinsic operational frequency approaching 200 MHz. These results provide strong evidence that the proposed RKKY-pMTJ architecture can achieve ultrafast, spin-torque-free response while maintaining thermal robustness, demonstrating a clear performance advantage over conventional in-plane TMR sensors.

The immediate future work will focus on the microfabrication and electrical characterization of TMR elements using the optimized stack. This crucial phase involves evaluating key electrical metrics—specifically the TMR ratio and sensor linearity—under various conditions. Most critically, robust performance must be validated across a wide range of temperatures through thermal cycling tests (e.g., −40 °C to +80 °C) to confirm commercial viability. Successful execution will pave the way for the deployment of these scalable, GHz-capable sensors for advanced electric vehicle and factory automation systems.

In future work, it will be necessary to fabricate a sub-100 nm-scale RKKY-pMTJ-based TMR sensor array, followed by full packaging with top and bottom electrodes, in order to systematically evaluate the electrical and magnetic properties of the TMR sensor devices.

## Figures and Tables

**Table 1:** Material parameters for micromagnetic simulations.

Material Parameter (Unit)	Reference Layer 1 (RL1)	Storage Layer (SL)
Saturation Magnetization (*M*_s_) (kA/m)	1300	1313
Perpendicular Magnetic Anisotropy Energy Density (*K*_u_) (MJ/m^3^)	1300	1300
Exchange Stiffness (*A*_ex_) (pJ/m)	15	15
RKKY Exchange Coefficient (*J*_RKKY_) (mJ/m^2^)	−1.2	

**Table 2:** Comparison of fabrication tolerance and scalability among MR sensor types.

Sensor Type	Typical Functional Layer (nm)	Thickness Tolerance for Functional Layer (%)	Scaling Potential	Limitation/ Advantage
AMR [6]	25	±10% (~±2.5 nm)	>1 µm	Signal weakens at nanoscale
GMR [6]	2.0 (spacer layer)	±5% (±0.1 nm)	≈200 nm	Interface roughness sensitive
In-plane TMR [6]	1.0 (MgO Barrier)	±3% (±0.03 nm)	≈100 nm	Barrier oxidation
Proposed RKKY-pMTJ	0.6 (Ru layer)	±17% (±0.1 nm)	<100 nm	IEC-based self-restoring, robust to scaling

**Table 3:** Switching times of *t*_SAF→SF_ and *t*_SF→SAF_ for applying external out-of-plane magnetic field (*H*_z_) pulses.

Switching Time	*H*z = 400 mT	350 mT	300 mT	250 mT	200 mT
*t*_SAF→SF_	1.39 ns	1.82 ns	2.59 ns	2.90 ns	2.94 ns
*t*_SF→SAF_	5.99 ns	5.96 ns	5.91 ns	5.84 ns	5.78 ns

## Data Availability

The data that support the findings of this study are available from the corresponding author upon reasonable request.
